# Learning about the nature of science through the critical and reflective reading of news on the COVID-19 pandemic

**DOI:** 10.1007/s11422-021-10092-2

**Published:** 2021-12-01

**Authors:** Antonio García-Carmona

**Affiliations:** Departamento de Didáctica de las Ciencias Experimentales y Sociales, Facultad de Ciencias de la Educación, C/ Pirotecnia S/N, Universidad de Sevilla, 41013 Seville, Spain

**Keywords:** Coronavirus, Nature of science, Pandemic, Science education, Science news, Socioscientific issue, asuntos sociocientíficos, coronavirus, educación científica, naturaleza de la ciencia, noticias de ciencia, pandemia

## Abstract

The global COVID-19 pandemic caused by the SARS-CoV-2 virus has led to a plethora of information published in the media. Conceived as a socioscientific issue of great relevance currently, this article highlight the educational potential of some media news about the pandemic to reflect and learn about the nature of science (NOS). To this end, a theoretical foundation is first presented regarding the reading of science news published in the media as an educational resource to learn about NOS. Secondly, a proposal is presented on how this might be addressed in the science classroom. The proposal is illustrated by four news items, intentionally selected as examples, which have been published in the Spanish digital press. The aspects of NOS that are discussed in the context of the news items selected are: tentativeness of scientific knowledge, role of error in scientific research, role of debate in the development of science, importance of models and modelling in scientific research, and ethics in science.

## Abstract (es)

La pandemia mundial de la COVID-19, provocada por el virus SARS-CoV-2, ha dado lugar a una gran cantidad de información publicada en los medios. Concebida como problemática sociocientífica de relevancia en la actualidad, este artículo destaca el potencial educativo de algunas noticias de los medios sobre la pandemia para reflexionar y aprender sobre naturaleza de la ciencia (NOS). Para ello, en primer lugar, se expone una fundamentación teórica relativa a la lectura de noticias de ciencia publicadas en los medios como recurso didáctico para aprender sobre NOS. En segundo lugar, se presenta una propuesta de cómo se podría abordar esto en clases de ciencia. La propuesta se ilustra mediante cuatro noticias, seleccionadas intencionadamente y a modo de ejemplos, que fueron publicadas en la prensa digital española. Los aspectos de NOS que se discuten en el contexto de las noticias son: provisionalidad del conocimiento científico, importancia de los modelos y la modelización en la investigación científica, y la ética en la ciencia.

## Understanding the nature of science for scientific literacy in times of pandemic

The understanding of basic notions regarding the nature of science (NOS) is considered a core component for scientific literacy of the citizenry (McComas & Clough 2020). There are several reasons to support this educational goal, but one of the most relevant is to prepare citizens to critically and responsibly participate in public or social issues related to science (Driver et al. 1996). As Sadler et al. (2004) point out, “(…) interpretation and evaluation of conflicting evidence in a socioscientific context is influenced by a variety of factors related to NOS such as data interpretation and social interactions including individuals’ own articulation of personal beliefs and scientific knowledge” (p. 387). In addition, an informed understanding of NOS provides arguments, for instance, to combat the claims held by science denialists and pseudo-science promoters. Thus, while this kind of person bases their arguments in false beliefs and assumptions (Fackler 2021), knowledge of NOS tells that science is based on evidence and its theories must overcome many verification tests through rigorous evaluation processes before they are accepted by the scientific community.

Consequently, the robustness or scientific soundness of the arguments that citizens put forward about socioscientific issues depends largely on how comprehensively informed they are about NOS. However, it is found that students’ understanding of NOS is in general quite limited, as can be seen in the abundant research literature on the topic (García-Carmona et al. 2012). Likewise, some studies have shown that when students make opinions about science- and technology-based issues, they take into account NOS aspects to a much lesser extent than other factors such as personal values, morals/ethics, and social concerns (Bell & Lederman 2003). Therefore, the development of an informed understanding of NOS among students continues to be a major challenge in science education.

The need to improve citizens’ understanding of NOS has been highlighted with the worldwide COVID-19 pandemic caused by the SARS-CoV-2 virus, which has become one of the most significant socioscientific issues in our present historical time. This can be considered a socioscientific issue because, in addition to the cure of COVID-19 is a research question that constitutes an important scientific challenge, the prevention, spread and cure of this disease are having a significant emotional, social, ethics, economic and political impact on citizenship.

The COVID-19 pandemic has led to the proliferation of pseudo-scientists offering miracle cures for the disease, to people who deny the existence of the coronavirus (the so-called flat-earthers of COVID-19), anti-vaccinationists, etc. Likewise, many people do not quite understand, for example, why scientists are changing their criteria to face this pandemic in the light of new data and its reinterpretations, or that it is not possible to go faster to obtain an effective medical treatment for COVID-19, or that certain politico-economic decisions come into conflict with the recommendations of the scientific community. Certainly, an appropriate knowledge of how science works and how it influences or is influenced by society can help to manage all these issues critically and responsibly.

Likewise, the COVID-19 pandemic can provide an excellent opportunity to reflect upon and learn about NOS in the science classroom. In this case, special attention should be given to the information published in the media about this socioscientific issue. In fact, for a large number of citizens, these media constitute their primary sources of science information (Hodson 2008). As Costa-Sánchez and López-García (2020) state:

The need to explain to the citizens what is happening [in relation to the coronavirus] and what the risks are, as well as involving them in the solution, make communication an important ally of the political, social, institutional, and health management of any such situation of this type. (p. 2; brackets added)

It could be said, therefore, that a person has an appropriate level of scientific literacy if they are able to understand science news published in major newspapers in their country (Miller 2004). And, as pointed out above, this requires, among other things, the ability to handle knowledge of NOS. With this in mind, the present article is aimed at describing how some news items related to COVID-19 could be used in the science classroom in order to reflect and learn about some aspects of NOS.

## Learning about NOS with science news published in the media

Literature on research in science education shows that discussions about socioscientific issues constitute an ideal opportunity for enhancing students’ understanding of NOS (e.g., Eastwood et al. 2012). According to Zeidler and Nichols (2009), socioscientific issues are controversial in nature; therefore, their approach in the science classroom implies the intentional use of science themes that demand students to engage in discussions and debates. These discussions usually require, among other factors, students to handle and become aware of aspects of NOS (Sadler et al. 2004), such as the limitations of science, the tentativeness of scientific knowledge, the possibility of interpreting a date in different ways, or the importance of scientific evidence.

Similarly, critical analyses of emerging scientific issues, which usually involve debates or some controversy within the scientific community, constitute a good approach to reflect and learn about NOS (García-Carmona & Acevedo 2016). According to Kipnis (2001), these analyses provide opportunities to tackle NOS aspects such as multiple theoretical interpretation of phenomena, choosing a theory, or insistence on the chosen theory. In the same vein, McMullin (1987) notes that scientific controversies (or incipient scientific issues) reveal the confrontation of different methodological and ontological aspects, which are underlying in any research activity. In addition, the analysis of emerging scientific issues helps in understanding that scientists’ work is also influenced by extra-scientific (or non-epistemic) factors that can condition the development of investigations, such as emotional, institutional, political, and economic pressures, interstate rivalry, and scientific fraud (García-Carmona & Acevedo-Díaz 2017, 2018).

An interesting resource to address all these aspects related to NOS consists of the reading of science news published in mass media, especially those which are published in digital press because of its easy access, in general, by most citizens. The reflective reading of science news from the media to discuss aspects of NOS has been highlighted in the science teaching literature (Shibley 2003). The first thing that should be taken into account is that such news items do not constitute a teaching resource per se since they were not written with the aim of being taught in the classroom. Therefore, it is important to first examine what their didactic potential is (García-Carmona 2014), i.e., to assess whether the scientific content covered in a news item is accessible for the students to whom it will be taught in classroom, whether it is conducive to reflection on certain aspects of NOS, whether it can be easily integrated with other content and activities of the science subject program, etc. Likewise, although the news from prestigious media can in general be considered to be reliable because its authors usually turn to specialized scientific sources (López-Pérez & Olvera-Lobo 2015), it is not exempt from certain biases. Fundamentally, there are two reasons for this (García-Carmona 2021a): (i) the interpretation and/or journalistic intention itself in publishing that news, and (ii) the possible simplifications or lack of rigour in its exposition due to trying to make the message understandable for the readers, whatever their level of scientific competence. In this sense, Costa-Sánchez and López-García (2020) have found examples of alarmism and sensationalism in journalistic reporting concerning the COVID-19 pandemic.

While numerous articles regarding the focus on the COVID-19 pandemic in science education have been recently published (e.g., Archila et al. 2021), very few of these suggest addressing NOS aspects despite being an ideal context to reflect and learn about them (e.g., Maia et al. 2021). In addition, few articles have been found that propose to address NOS aspects in the science classroom using news items related to the COVID-19 pandemic published in digital press (e.g., Demirdöğen & Aydın-Günbatar 2021). Therefore, this manuscript has been developed to contribute to the latter through a proposal based on this didactical framework, as will be seen below.

### What aspects of NOS can be addressed with science news in the media

When selecting news items from the media to address aspects of NOS in the science classroom, three possibilities can be considered: (i) search for science news that allows aspects of NOS previously set in the learning objectives to be dealt with, irrespective of the science content, (ii) search for science news related to a specific topic or content of the school science curriculum, and see what aspect of the NOS could be addressed with it, or (iii) search for news about a specific school science topic or content that also allows aspects of NOS previously set in the learning objectives to be dealt with. The present article used the second criterion, i.e., the premise of the search was to locate news in the media about the coronavirus, and then assess which NOS topics could be covered.

In a review of news items about physics that were published in Spanish daily newspapers during the period 2006–2013, García-Carmona (2014) found that they could be grouped into three types according to the aspects covered: advances and limits of science (e.g., new experiments and discoveries, debates in the scientific community), history of science (e.g., ephemerides of scientific milestones and relevant people in science), and contemporary socioscientific controversies. The most abundant type of news was that which referred to the advances and limits of science. This highlights the fact that science news in the media is a valuable resource in discussing in the classroom the ongoing real-time dynamism of science.

It should be highlighted that NOS is *meta-knowledge* of how science works and progresses, which is built mainly with contributions from the philosophy, history, and sociology of science. Therefore, it is a polyhedral and dynamic construct that can admit diverse conceptualizations. In the literature there are various proposals regarding what to teach about NOS, although this present article will not be about comparing and analysing such proposals. However, it must be said that the proposal of NOS content put forward by Lederman (2007) is one that has received more attention in the international panorama over the last two decades. His proposal is essentially circumscribed to the understanding of epistemic features of science (laws vs. theories in science, observation vs. inference, tentative nature of scientific knowledge, methodological plurality of science, etc.). In this, only a generic reference is made to the notion that the construction of scientific knowledge is influenced by the social context, and vice versa. But, when the history, philosophy, and sociology of science is reviewed, it is found that multiple non-epistemic factors, such as contextual, social, economic, political, and psychological ones, also decisively influence its development (García-Carmona 2021b). In addition, it can be observed that the development of scientific research related to COVID-19 is clearly replete with epistemic and non-epistemic aspects. Consequently, the position adopted here will be one that covers both types of NOS aspects. Logically, underlying this framework proposal is the idea that it is science educators (depending on their pedagogical content knowledge, the school context, and the educational goals set out in their science program) who will be the ones selecting the most appropriate aspects of NOS to address at each moment or in each lesson. In the proposal that will be described here, discussion is promoted regarding the following NOS aspects: tentativeness of scientific knowledge, role of error in scientific research, role of debate in the development of science, importance of models and modelling in scientific research, and ethics in science.

### How to address aspects of NOS in the context of science news in the media

The integration of NOS content into the school science curriculum should not result in the mere promotion of certain pieces of declarative and generic knowledge about the characteristic features of scientific knowledge and activity. Rather, attention in the science classroom should be given to promoting students’ critical and reflective analysis, by contrasting their own ideas with those coming from the philosophy, history, and sociology of science. This type of analysis should involve the students to perform diverse practices such as inference, reasoning, comparison, argumentation, critique, and explanation. All of these are essential for the development of *critical thinking* (Dwyer et al. 2014), which is conceived as a foundational pillar to learn about NOS (Yacoubian 2020). Therefore, as indicated by educational research in this regard, the best way to approach the teaching of NOS in the classroom is explicitly, i.e., with specific learning objectives and the design of activities for the students to think about and discuss aspects of NOS, and an assessment process to determine the degree of understanding they have attained (García-Carmona & Acevedo-Díaz 2018).

As mentioned above, the analysis of news from the media about science has been highlighted as an ideal resource with which to promote an educational perspective of discussing and learning aspects of NOS. One of the strengths of this resource, with an appropriate selection of the news items, is that it allows a NOS aspect to be dealt with in an integrated or contextualized way together with the content of the school science curriculum. In agreement with Clough (2006) and Allchin (2011) among other authors, the introduction of aspects related to NOS in authentic contexts of scientific development, (socio)scientific controversies, etc., encourages students to gain a more realistic view of the activity and repercussions of science. This will help them to see the sense of discussion about aspects of NOS and to transfer their understanding of these aspects to other situations related to the development of science. Likewise, the integration or contextualization of aspects of NOS with other content of the science curriculum has the advantage that it would scarcely alter the programming planned for a science course (Bell et al. 2012).

However, in line with what Oliveras et al. (2013) say, the message that a science news item wishes to put out to society is not always clear to all readers, and possibly neither do they all interpret it in the same way. Each reader will identify and interpret the message in accordance with their own scientific background. Similarly, the content of a science news item can be conducive to discussing certain issues that perhaps the author had not even considered emphasizing. Therefore, reading science news in the classroom, both in general and particularly when dealing with NOS issues, should be accompanied by a series of questions that are specific and contextualized in the content of that news, so as to focus critical reflection on those aspects of NOS that it is the aim to discuss (García-Carmona & Acevedo 2016).

In accordance with the above, the following paragraphs illustrate, by way of some examples of news from the digital press related to COVID-19, how some aspects of NOS could be discussed in the classroom by taking a reflective approach.

## The COVID-19 pandemic in the digital press: Some examples to address aspects of NOS

In what follows, four examples will be developed of how to deal with certain aspects of NOS in the context of news items published in the Spanish digital press during the COVID-19 pandemic. By way of clarification, since the purpose is to describe how aspects of NOS might be addressed in the classroom in the context of this socioscientific issue, no systematic selection of the news was made. Instead, various news items were deliberately selected which, in the opinion of the author of this article, were conducive to discussing some aspects of NOS. In this case, they are the tentativeness of scientific knowledge, the role of error in scientific research, the importance of models and modelling in scientific research, and the role of debate in the development of science as epistemic aspects, and ethics in science as non-epistemic. It should be noted that ‘debate’ is considered here as a purely epistemic aspect of NOS because, as will be seen below, what is exposed in the news item selected to deal with it is a rational discussion among scientists who show different conclusions on a particular natural phenomenon.

The four news items were published in the digital editions of the following three Spanish newspapers:

- *El País (elpais.com)*: According to the Socialscene report prepared by Apple Tree Communications (Available at https://www.appletreecommunications.com/wp-content/uploads/2017/07/socialscene-july-2017-eng.pdf), it is the Spanish-language digital newspaper most followed in the world. This is also edited in other languages, among which is English. It is included in the international top 100 media by a number of followers, and it is the second most read newspaper in Spain (the first is a sports newspaper) according to a recent study on digital market (available at https://es.statista.com/estadisticas/476795/periodicos-diarios-mas-leidos-en-espana/). The newspaper publishes all type of news (politics, society, economy, culture, sports, etc.) including a section called “science”.
- *ABC (ABC.es)*: it is the oldest newspaper in Spain. This also publishes all types of news (politics, society, economy, culture, sports, etc.) including a “science” section. This newspaper is only edited in Spanish, and according to the recent study on the digital market, it is among the four most widely read non-sports newspapers in Spain.
- *Eldiario.es*: This is a digital newspaper founded much more recently than the other two. It is only edited in Spanish and basically publishes news related to politics, society, and the economy. Therefore, it does not include a specific section dedicated to science, but it also publishes some science news items when the issues addressed are of great interest for society.

It must be pointed out that reading of news items published in the media to reflect on the aspects of NOS could be started as early as middle school (García-Carmona, 2014). However, this will depend on each educational context and above all on how familiar students are with this educational resource (i.e., the critical and reflective reading of science news). The resource is also conducive and effective in the training of prospective primary teachers on NOS (García-Carmona & Acevedo 2016).

### Example 1: science is in permanent revision, and controversies are frequent in the scientific community

On 7 April 2020, the Spanish digital newspaper *eldiario.es* published an article with the following title or headline: "*An unsolved scientific debate: Is the coronavirus a living being?*" (by Enrique Sacristán; available at https://www.eldiario.es/sociedad/coronavirus-explicar_0_1013848704.html). The content of this news item makes it conducive for reflection on the tentative and dynamic nature of scientific knowledge, as well as on the importance of debate in the scientific community so as to establish theoretical knowledge. In the subtitle of the item, one can read the following:

Scientists disagree when asked whether a virus that has infected more than a million people around the world is a living being. Some consider that this “robot” of RNA and proteins is not a living being.

Likewise, the body of the news item includes such fragments as the following:

[The coronavirus] reaches a human cell through the mucosa, attaches itself to a specific component of its membrane, makes a hole and enters, introduces its chain of genes into the cellular mechanism and “tricks” it into producing the components of new viruses, which end up looking for further victims.

Is something that does this alive? "I think so," says [Margarita] Del Val, "but I am aware that the opinion of other virologists is different, in particular those who work and reflect on the origin of life. Their commonest objection is that viruses are never metabolically active, this being a vital characteristic together with those of multiplying and evolving ".

[Ester Lázaro]: "When I started working with viruses more than 20 years ago, it was difficult for me to believe that there were those who doubted whether they were living beings or not,” she comments. “After all, they are made of the same molecules as life, including a genome in which information about its properties and functioning is stored. (…) ".

Carlos Briones, also a researcher (…): "Our current knowledge supports the idea that viruses and viroids (even simpler infectious agents) should not be regarded as living beings, although they are fundamental in the evolution of life and in the configuration of our biosphere.

[Ester Lázaro]: "Although I think that viruses are not living beings, they are not like inert matter either,” she admits. “The debate is still open and very likely it will continue like this for a long time. Perhaps we should give up categorizing and limiting, and accept that between life and non-life there are entities that we do not know very well how to classify but that fulfil their function in the history of life".

According to the information provided by the news item, the following could be some questions with which to reflect on the aspects of NOS indicated:

1. According to what you have read in the news item, there are scientists who consider viruses to be living beings and others who do not. What do you think usually happens in the scientific community to resolve debates like this?
2. One of the scientists interviewed talks about how her conception of a living being has changed over the years. (a) What factor or factors do you think may have influenced this change of conception of living being? (b) How do you think this change in conception may have influenced this scientist’s research work?
3. What interest or importance do you think there is for the scientific virologist community to determine whether or not viruses are living beings?

### Example 2: the role of error in the development of science

Concern about the COVID-19 pandemic has led to a certain scepticism on the part of the public regarding the work of scientists, because they are seen to change their criteria, health recommendations, etc. This has encouraged some scientists to explain in the media what scientific activity consists of while conducting an investigation and how errors are an inherent part of this, and therefore, inevitable in the work of science. For example, on 25 May 2020, in the Spanish digital newspaper *elpais.com* an article was published with a quite revealing title: "*Scientists do not stop being wrong*" (by Pablo G. Pérez and Patricia Sánchez; available at https://elpais.com/ciencia/2020-05-25/los-cientificos-no-paran-de-equivocarse.html). In the first paragraph of the news item, one can read:

Two months ago, we heard things like "you have to invest in science" or "research is essential to defeat the pandemic". But it was also put about that "the data are bad", "not even scientists know what to do", "they are deceiving us with the data"... Unfortunately, now we have reached this stage of "the scientists are deceiving us" (...).

Further on, in an attempt to explain how errors and mistakes are handled in the development of science, the authors of the article write:

More than 50% of a good researcher’s time is spent in seeing where they have gone wrong. Scientific [work] is based on a critical and sceptical spirit, but one that is also constructive and reasoned, and the certainty is that the most probable thing that can happen when taking data, analysing it, interpreting it, and putting it out in public is that you will make a mistake. (…) a large part of our work as scientists is to devise checks, tests, and experiments (…) to detect the many mistakes we may have made. And when we can’t think of any more tests and our interpretation of the data has survived all of them, we can say that perhaps we have got a result (…).

This is how science usually advances. Only error leads to knowledge. With data that are not usually wrong or misleading, but that are always limited, and therefore partial and biased, and with a critical, objective, and truthful discussion about the benefits, defects, and limitations of interpretive theories, we advance in understanding the problems. For scientific disciplines such as medicine, this is tragic, very sad, and disturbing. But if science faces the unknown (…), to err will be normal.

Therefore, the content of this news item is ideal for reflection on the role or incidence of error in the development of science. For this, the following questions could be posed (García-Carmona & Acevedo-Díaz 2018):

1. Based on what you have read, how do you think scientists become aware that they have made a mistake in their research?
2. What do you think scientists do to avoid errors in their research?
3. What do you think scientists do when they detect errors in their research?
4. What do you think about the following statement: "The best scientists are those who do not make errors in their research"?
5. If scientists make errors in their research, how do you think this influences the development of science?

### Example 3: the role of models in the development of science

Another important scientific aspect in the fight against the coronavirus is the elaboration and use of models to explain and predict its behaviour. Numerous news items have been published in the Spanish media in this regard. An example of these is the one published on 7 April 2020 in the newspaper *ABC.es* under the following headline: "*The three mathematical methods that are saving lives from the coronavirus*" (by Gonzalo López; available at https://www.abc.es/ciencia/abci-tres-metodos-matematicos-estan-salvando-vidas-frente-coronavirus-202004062243_noticia.html). This news item is quite interesting for fostering classroom reflection on the role of scientific models and their nature. In the body of the item one can read excerpts like this:

Several mathematicians and epidemiologists have (…) explained how, despite having incomplete information, the models can reconstruct the "submerged part of the iceberg".

In addition to surveys and statistics, mathematical models are being used to fight COVID-19. In general, they try to extract the aspects that are relevant for a real situation and translate them into the form of mathematical equations to forecast how certain measures or factors will affect its evolution.

Thanks to this model [SEAIHR], important predictions can be made. For example, (…) study how to lift confinement measures progressively, and perhaps focusing on certain age groups such as the elderly.

Some questions that could be posed to the students to encourage such reflection on scientific models are the following:

1. Based on what you have read in the news item, why do you think scientists make models of the phenomena they study?
2. What do you think about the fact that there are several scientific models of the same phenomenon?
3. Analyse the following statement: "Scientific models represent the objects or phenomena to which they refer as they are."
4. In general, what advantages and limitations do you think the use of models in scientific research has?

### Example 4: ethical aspects in the development of science

The COVID-19 pandemic has also generated discussion about some ethical aspects related to scientific research. The fact of wanting to obtain a vaccine quickly is calling into question whether the steps that are being followed in the process are maintaining consistency with the ethical codes of medical research. This matter has had an impact in the Spanish digital press. For example, on 3 April 2020, the following news item was published in the newspaper *ABC.es*: "*The world faces the serious dilemma of skipping crucial steps in the development of a vaccine against COVID-19*" (by José Manuel Nieves; available at https://www.abc.es/ciencia/abci-mundo-enfrenta-grave-dilema-saltarse-pasos-cruciales-desarrollo-vacuna-contra-covid-19-202004022037_noticia.html). In this news item, one can read, among other things, the following:

Many laboratories around the world are these days fighting in a race against the clock to obtain a vaccine with which to stop the coronavirus pandemic (…). But proving that a vaccine works safely can be painfully time consuming.

(…) at the same time as the pandemic is advancing at a pace that very few could have imagined, a provocative and ethically complicated proposal is making space for itself which consists in eliminating several of the usual steps in the development of vaccines and saving many months of time.

"People who are faced with a terrifying problem like this," says the scientist, "will more easily opt for unusual measures. We have to rethink our prejudices."

(…) Seema Shah, a bioethicist at Northwestern University (…) has serious doubts, [and] says that the ethical scales could be tilted in favour of these experiments if the volunteers were people already prepared to take certain risks, such as health workers. "The public," says the researcher, "is not familiar with these kinds of trials, which sound completely contradictory and contrary to the standard notion of what doctors and researchers are supposed to do."

Discussion about ethical aspects related to scientific research, in the context of this news item, could be generated with the following questions:

1. After reading the news item, why do you think it is necessary to have ethical codes for the different fields of scientific research?
2. In what way do you think the ethical beliefs and values of scientists can influence their research?
3. How do you think ethical codes are developed in scientific research? What criteria do you think the scientific community has to elaborate those codes?

## Final comments

As was advanced at the beginning, the understanding of aspects of NOS should constitute a key pillar in citizens’ scientific literacy. Addressing this understanding in science education can provide students with informed ideas about how science works, so as to critically analyse information published in the media about socioscientific issues, such as those related to the COVID-19 pandemic.

This present article has argued that a good way to promote such informed knowledge about NOS so as to digest all this information is precisely to use the media themselves – in this case, the digital media. To illustrate this, a proposal has been presented, through four examples, for how certain aspects of NOS could be dealt with in the classroom in the context of daily news items in the digital press that are related to or directly address COVID-19. The proposal is designed so that informed knowledge about NOS can be developed through an analysis that requires (i) an initial personal reflection by students about the questions raised in the context of the news item to enable them to express their own ideas about the aspects of NOS being dealt with; (ii) a debate or discussion of those ideas with classmates to determine their similarities and differences; (iii) a search for a consensus of ideas about the different aspects of NOS being discussed; and (iv) confrontation of those consensus ideas with theoretical frameworks in the literature related to notions about NOS. All this must be orchestrated by the educator, who must stimulate discussion, introduce nuances, or raise auxiliary questions so as to enrich the debate and to generate cognitive conflict when misinformed conceptions are exposed so that it is the students themselves who build their own knowledge about NOS. In order to illustrate how to implement all of this in the science classroom for each news item, the following three phases are suggested (adapted from García-Carmona 2021a):

- *First phase: Reading the news item.* Without prior instruction and organized in small work groups, the students read the news and answer the questions for reflection about the aspect(s) of NOS selected. The educator encourages their answers to be the result of a previous discussion and the consequent consensus among all the group members. Nevertheless, it if contrary opinions appear, making consensus impossible in the answer, then the different opinions can be expressed.
- *Intermediate phase: Whole-class discussion of initial answers.* After answering the questions put to them, the groups share and discuss their views in classroom. To this end, a representative of each group presents the answers of her/his group to the others. After all the groups present their answers, a question time and debate should be stimulated. The educator should moderate the discussion among the groups and make clarifications, raise additional questions to deepen and/or redirect the discussion, etc., all this with the aim of enriching the sharing as much as possible. When the students present weakly informed ideas about the topic being analysed, the educator should try to elicit cognitive dissonance so that they reconsider their arguments. The aim is to encourage the groups to reach common conclusions about the aspect(s) of NOS dealt with.
- *Final phase: Conclusions after the whole-class sharing session.* After the discussion among the groups about the questions they have been set, each group must review their initial answers (first phase), introducing all those corrections, qualifications, or extensions they consider necessary to improve their arguments and ideas regarding the aspect(s) of NOS discussed.

On the other hand, although the news items used to illustrate the proposal were published in the Spanish digital press, there are sure to be similar news items that have been published in the digital newspapers of other countries with different languages. Therefore, the approaches described here can be easily extrapolated to other contexts by selecting the news items most conducive to reflection on the desired aspects of NOS.

In the near future, it is expected that the reading of news items such as those listed above will be implemented in the science classroom, and their educational effectiveness assessed, in order to reflect and learn about the aspects of NOS discussed herein. However, it was deemed timely to share this educational resource in advance with the science teaching community so as to address the socioscientific problems associated with the pandemic through the lens of NOS.
